# Slow dynamics of human balance control

**DOI:** 10.1038/s41598-025-09392-2

**Published:** 2025-07-29

**Authors:** Kyle J. Missen, Mark G. Carpenter, Lorenz Assländer

**Affiliations:** 1 School of Kinesiology, University of British Columbia, Vancouver, Canada; 2 International Collaboration on Repair Discoveries, University of British Columbia, Vancouver, Canada; 3 Djavad Mowafaghian Centre for Brain Health, University of British Columbia, Vancouver, Canada; 4 Department of Sport Science, Human Performance Research Centre, University of Konstanz, Konstanz, Germany

**Keywords:** Postural control, Sensory integration, Torque feedback, Balance control model, Self-calibration, Motor control, Sensorimotor processing

## Abstract

When standing on a tilting surface, humans’ sway behavior at frequencies below 0.1 Hz indicates the contribution of a slow feedback component. We suggest this may reflect a self-calibration mechanism of the balance control system, constantly referencing orientation estimates based on kinematic sensory cues to a reference based on force cues. However, attempts to identify this mechanism have been limited by insufficient experimental trial durations and small sample sizes. This study aimed to assess the properties of the mechanism that reduces body sway at very low frequencies in upright standing. Anteroposterior body sway responses to short- and long-duration surface tilts were measured and interpreted using balance control models. Four feedback control model variants, with different mechanisms to account for the slow dynamics, were fit to experimental data. Furthermore, we tested how estimates of the slow component are affected by stimulus period duration. We hypothesized that the model variants containing force cues would provide the best fit to experimental sway responses, particularly in response to long-duration surface tilts. Our results confirm this hypothesis and suggest that humans use integrated force afferents from the feet and legs in a slow, positive feedback mechanism during standing to remain upright. Despite stimulus period durations of ~ 180 s, some properties of this mechanism were difficult to estimate. The positive torque feedback mechanism aligns with the notion of self-calibration.

**Supplementary Information:**

The online version contains supplementary material available at 10.1038/s41598-025-09392-2.

## Introduction

Kinematic sensory cues from visual, vestibular, and proprioceptive systems are combined within the nervous system^1^ to form an internal representation of body orientation and motion in space. This representation guides the central nervous system in generating the appropriate joint torques needed to maintain an upright standing position. However, sensory cues are inherently noisy, as the relationship between physical events and transducer firing rates constantly changes^2,3^, and central processes such as integration and differentiation can introduce signal drift. As a result, there is considerable uncertainty in the information upon which this internal representation is constructed. Without an ‘absolute’ reference for noisy sensory estimates, the balance control system is susceptible to instability. Thus, the balance system requires a continuous ‘self-calibration’ mechanism to accurately estimate body orientation in the presence of sensory noise and uncertainty.

A self-calibration mechanism that is robust to noisy sensory cues should, conceptually, operate on a relatively slow timescale. This would enable the balance control system to filter out rapid, noise-related sensory fluctuations and allow time for recalibrating changes in body orientation without instability. There are two empirical observations indicating the existence of a sensory feedback mechanism contributing to slow, low-frequency sway. First, sway responses to surface or visual scene tilts at frequencies below 0.05 Hz are smaller than would be expected of a feedback mechanism without a low-frequency component^1,4–7^. Second, when experimentally blocking body movement in space while participants continue to actively control their balance, the center of pressure (COP) exhibits a slow drift, with participants generating considerable ankle plantarflexion torque over time^8,9^. This effect can be amplified by slightly tilting the participant after blocking body movement, which causes them to generate a small torque response. This small torque response leads to a systematic drift in the COP, indicating a self-amplification of torque^10^.

Balance control models often represent the slow component using a sensory torque feedback mechanism^5,6,11–13^. This mechanism uses low-pass filtered torque afferents from the central processing of sensory foot pressure and muscle force receptors and is organized as a positive feedback loop nested within the negative feedback control of balance^6^. The functional outcome of low-pass filtered positive feedback is a mechanism that reduces the effort needed to stand and helps align the body with the gravito-inertial force^7^. Others have represented the slow component in balance control models by substituting the torque feedback loop with the integral term in a proportional-integral-derivative (PID) controller^1,14–16^. The output of the integral term is proportional to a running sum of errors (deviations from upright) over time, which minimizes slow angular biases from the upright reference. Due to their simplicity and versatility, PID controllers are frequently used in engineering to represent closed-loop processes. Applied to balance control models, the PID controller integrates kinematic sensory errors over time, gradually bringing the system to an upright position.

The PID controller and torque feedback mechanism describe behavior similarly at frequencies above 0.05 Hz and can be used interchangeably to fit experimental sway responses to relatively short trial durations^17^. At frequencies below 0.05 Hz, a model incorporating torque feedback demonstrates a better fit to experimental sway responses than a PID controller. Specifically, the torque feedback model exhibits a phase lead consistent with observed experimental responses, while the phase of the PID controller tends to approach 0°^6^. However, it is important to note that the model fits from Peterka^6^ were based on experimental data from a single participant with bilateral vestibular loss. Furthermore, this comparison was based on a variant of the torque feedback mechanism that integrates torque over an infinite amount of time. Because biological processes cannot integrate indefinitely, alternative versions of the torque feedback loop are formulated as low-pass filters consisting of a time constant with a finite time horizon (i.e., leaky integrators)^5,10–13^. Previous research has attempted to estimate this integration time horizon but observed highly skewed estimates, indicating limited accuracy^12^. Subsequently, the torque feedback time constant properties are often assumed from previous estimates and excluded from parameter estimation procedures^11,13^, resulting in an incomplete understanding of this mechanism. In summary, there is a lack of experimental data to differentiate between alternative formulations of the low-frequency mechanism of human balance control and to better identify its properties.

As indicated by the findings of Peterka^6^, evidence to determine which model best represents human balance control may be revealed by observing behavior over extended periods. Previous studies have described the properties of the slow mechanisms using stimulus sequence lengths between 45 and 60 s but have observed inaccurate and skewed parameter distributions^12^ and highly variable parameter estimates^1^. To our knowledge, no experiments have directly compared these slow mechanisms with stimulus sequence lengths beyond ~ 1 min. Additionally, the potential benefits of stimulus sequences exceeding ~ 1 min on the ability to distinguish the mechanisms described above and on the accuracy of model parameter estimates, particularly those associated with the low-frequency balance components, remain unclear.

This study aimed to assess the properties of the mechanism that reduces sway at very low frequencies in upright standing. Anteroposterior body sway responses to 60.5-s and 182-s long support surface tilts were measured, and balance control models were fit to the response characteristics. Four model variants, varying in complexity and their interpretation of the slow dynamics, were fit to experimental data. We hypothesized that model variants incorporating torque feedback would provide the best fit, specifically in the low-frequency range. We further hypothesized that longer stimulus periods would lead to more accurate estimates of model parameters related to the slow dynamics of balance control.

## Methods

A subset of the data in this study was previously published in a study examining the velocity dependence of the sensory reweighting mechanism for standing balance^18^. However, the primary analyses described herein have not been previously reported.

### Participants

Twenty healthy young adults (10 females, mean ± standard deviation age = 24.2 ± 2.7 years, height = 1.8 ± 0.1 m, weight = 74.4 ± 13.2 kg) participated in this study. None of the participants had neurological and/or musculoskeletal disorders that could affect their balance. Written informed consent was obtained from participants before the experimental procedures, which were compliant with the Declaration of Helsinki and were approved by the University of Konstanz Ethics Board and the University of British Columbia Behavioural Research Ethics Board.

### Experimental procedures

Participants stood on a custom-built servo-controlled tilting platform. Feet were positioned at a self-selected stance width with ankles aligned with the platform’s axis of toes-up/down rotation. Two hooks, secured at hip and shoulder level using Velcro straps, guided horizontal rods affixed to potentiometers. Hip and shoulder sagittal plane sway angles measured by the horizontal rods were transformed to center of mass (COM) displacements via a 120-s calibration trial involving slow movements at the hip and ankle joints and trigonometric calculations (see ^17,19,20^). Finally, COM displacements were used in conjunction with estimates of COM height obtained from anthropometric tables to determine the COM sway angle^21^. Support surface and sway rod data were sampled at 1000 Hz.

Participants completed four experimental conditions in this study^18^; only data from two conditions are presented in the current analyses. In each condition, the support surface rotated following a pseudorandom ternary sequence (PRTS). A PRTS waveform drives the platform rotation in an unpredictable pattern and has the unique property of containing zero amplitude at all even frequencies. Details pertaining to the generation of PRTS stimuli can be found in ^1^. We designed a PRTS with a sequence length of 60.5 s (short-PRTS condition; *fmin* = 0.0165 Hz) and a PRTS with a sequence length of 182 s (long-PRTS condition; *fmin* = 0.0055 Hz). The velocity of the sequences (0.47°/s) was matched to keep expected sensory weights and other control parameters similar across conditions^18^. This resulted in peak-to-peak amplitudes of 2° for the short-PRTS sequence and 3.6° for the long-PRTS sequence. Short-PRTS trials consisted of four stimulus sequences (trial = 242 s), and long-PRTS trials consisted of two stimulus sequences (trial = 364 s). Each trial started with the final 20 s of the stimulus sequence, which was included to allow participants to reach a steady state and was discarded before analysis. Eight sequences of each stimulus were completed across two visits to improve the reliability of feedback control model parameter estimates^17^. Before the experimental trials on a given visit, participants completed a familiarization trial that consisted of a range of stimulus amplitudes and velocities.

All trials (including the familiarization trial) were completed with eyes closed. A nonrhythmic audiobook was played through over-ear headphones to avoid boredom^22^, distract from the balance task, and block equipment noise. Participants were instructed to stand upright and comfortable with their arms crossed in front of their hips before each trial. Seated rest periods were provided halfway through the experiment and at the participant’s request.

### Data analysis

#### Balance control system dynamics

Data analysis and model simulations were performed in MATLAB (The MathWorks, Natick, USA). The eight sequences of surface tilt and COM body sway data were transformed to the frequency domain using a fast Fourier transform and averaged to produce a single stimulus and body sway spectrum. The average body sway spectrum was divided by the average stimulus spectrum, and even frequencies (i.e., where the stimulus has no energy) were removed before calculating frequency response functions (FRFs). FRFs were represented as gain and phase, which reflect the ratio of COM body sway to tilt stimulus amplitude and their temporal relationship across frequencies. Coherence was also calculated as a signal (sway evoked by the stimulus) to noise (random sway component) measure (for details, see ^17^). FRFs were smoothed by averaging across adjacent frequencies to reduce variability and generate logarithmically spaced frequency points for model fits^1^. The FRFs used for further analysis contained 13 (0.016–1.46 Hz) and 14 (0.0055–1.46 Hz) frequency points for the short-PRTS and long-PRTS conditions, respectively.

#### Balance control models

Parametric interpretations of experimental FRFs were obtained using variants of the Independent Channel model^1^. The Independent Channel model is a widely used, relatively simple linear feedback control model that accounts for stimulus-dependent changes in FRFs through sensory reweighting. The basic components of this model represent known features of the balance control system, making it a useful model for understanding experimental results with physiologically relevant interpretations. In this model, the body mechanics are represented as a single-link inverted pendulum, which simplifies anteroposterior body motion equations and adequately captures a standing human’s behavior^23,24^. Body motion is encoded by visual, vestibular, and proprioceptive sensory systems, which are assigned a relative weight (*Wvis* + *Wprop* + *Wves* = 1) and combined to generate an internal estimate of body orientation. We measured participants with eyes closed, such that only proprioceptive and vestibular cues, as well as somatosensory force cues (see below), contribute to balance control (with eyes closed, *Wvis* becomes 0 and *Wprop* + *Wves* = 1). The internal estimate of body orientation is compared to an internal reference point that specifies a desired upright body position; differences between the body orientation and the internal reference produce a sensory error. The sensory error passes through a lumped time delay, accounting for sensory and motor transmission, processing, as well as muscle activation delays. The delayed sensory error is amplified by a neural controller to generate ankle torque.

#### Formulations of slow balance control component

**Fig. 1:**
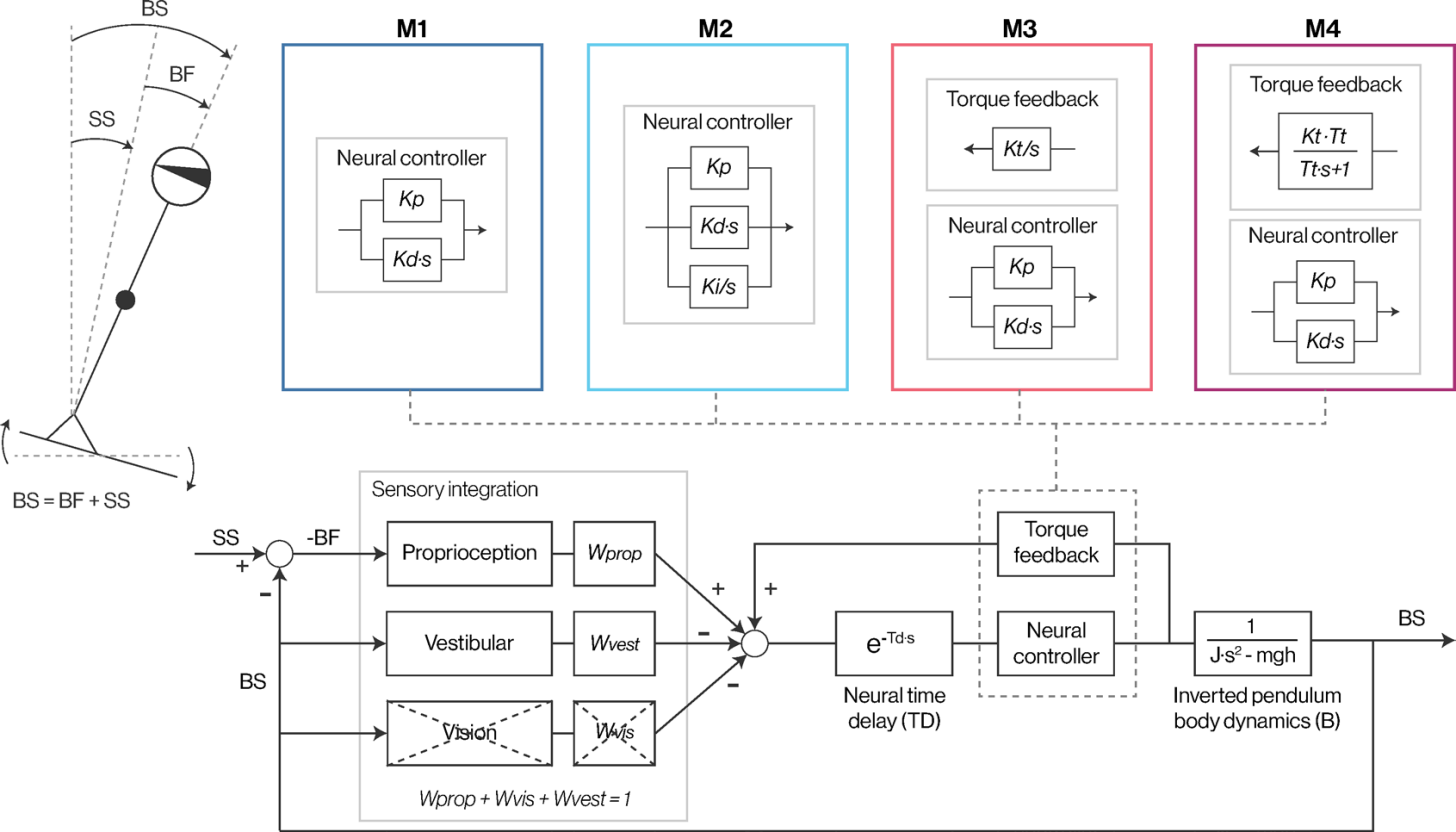
Balance control model schematic with variants. The body is controlled by a sensory integration mechanism with weighted sensory channels. The representation of neural controller and torque feedback elements varies depending on the specific model variant (M1, M2, M3, M4). SS, support surface angle; BS, body-space sway angle; BF, body-foot angle. See text for description of model parameters.

Four variants of the Independent Channel model were fit to experimental FRFs. The four variants differed in their mathematical formulation and interpretation of low-frequency balance control dynamics (Fig. 1).

The first variant, the PD controller (M1), represented the simplest variant and is the minimum control scheme required for stabilizing an inverted pendulum with a time delay^25^. The PD controller does not contain a slow component and does not account well for dynamics < 0.1 Hz^6^. M1 served as the basis for the remaining model variants, where different slow feedback mechanisms were added to M1. M1 is given by:

1 $$\frac{{\theta }_{bs}(s)}{{\theta }_{ss}(s)}=\frac{{W}_{prop}\cdot NC\cdot B\cdot TD}{1+NC\cdot B\cdot TD}$$

This transfer-function formulation gives the frequency-dependent ratio of body sway (${\theta }_{bs}(s))$ to support surface stimulus (${\theta }_{ss}(s))$ using the Laplace variable $s= i\cdot 2\uppi f$. The model contains the parameter *Wprop* as the relative contribution of proprioceptive information, NC = *Kp* + Kd*s as the proportional and derivative feedback parameters (proportional: amplifying the difference between desired and actual position; derivative: amplifying the derivative of this difference), *TD* = *e*^*-Td·s*^ as the lumped feedback time delay, and the body mechanics $B= \frac{1}{J\cdot {s}^{2}-mgh}$ representing the body as a single-link inverted pendulum using the small angle approximation sin($\theta$) ≈ $\theta$.

The second variant (M2) implements the slow component as an additional integral feedback component (i.e., assuming a PID controller). The integral component sums the sensory error over time. The Laplace equation for M2 is identical to that of M1 in Eq. (1), with the exception that *NC* = *Kp* + Kd*s + $\frac{Ki}{s}$.

The third variant (M3) adds torque feedback to M1. Three key properties distinguish this feedback from the kinematic feedback in the model. First, it relies on somatosensory force/torque cues rather than the kinematic feedback error used in the integral feedback of M2. Unlike kinematic sensory cues, these torque cues are not assigned relative weights. Second, torque feedback operates in a positive feedback manner, in contrast to the negative feedback mechanism of kinematic sensory cues. Third, as positive feedback leads to self-amplification, torque feedback must operate with very slow dynamics; otherwise, it would lead to system instability. In M3, the slow dynamics mechanism is implemented as an integral of torque cues with the positive feedback gain parameter, *Kt,* where $F= \frac{Kt}{s}$. The Laplace equation, including positive torque feedback, is given by:

2 $$\frac{{\theta }_{bs}(s)}{{\theta }_{ss}(s)}=\frac{{W}_{prop}\cdot NC\cdot B\cdot TD}{1-F\cdot NC\cdot TD+NC\cdot B\cdot TD}$$

The fourth variant (M4) resembles M3 but contains a leaky integrator, $F=\frac{{K}_{t}\cdot {T}_{t}}{{T}_{t}\cdot s+1}$ with the time constant *Tt* and the gain parameter *Kt*. The gain parameter Kt represents the extent to which low-frequency sway is reduced by torque feedback, with higher values reflecting stronger reductions, whereas the time constant represents the memory of the torque feedback (i.e. the integration is only across the corresponding time horizon). Thus, for very long time constants (approaching infinity), M3 and M4 are equivalent.

#### Parameter estimation

Anthropometric tables^21^ were used in conjunction with participants’ height and weight to estimate body inertia, body mass (excluding the feet), and COM height above the ankle for each participant. Parameters for each model variant were estimated for each participant and condition using a global optimization procedure (MATLAB Global Optimization Toolbox), which minimizes the normalized error between sequence-averaged experimental FRFs and simulated FRFs. The objective function was given by:

3 $$err= {\sum }_{i=0}^{n}{\left(\frac{\left|{FRF}_{sim}({f}_{i})-{FRF}_{exp}({f}_{i})\right|}{\left|{FRF}_{sim}({f}_{i})\right|}\right)}^{2}$$

where FRF_sim_ represents the FRFs obtained from simulations and FRF_exp_ represents experimental FRFs. As described above, the model variants contained different numbers of parameters, but four parameters were common to all model variants. Thus, we classified model parameters as model-independent (*Kp, Kd, Td, Wprop*) and model-dependent, denoting their corresponding model variant with subscripts (M2: *Ki*_*2*_; M3: *Kt*_*3*_; M4: *Kt*_*4*_ and *Tt*_*4*_).

#### Parameter confidence bounds

A bootstrap analysis was performed to determine how longer-duration stimulus cycles influence model parameter confidence bounds. Eight cycles of Fourier-transformed surface tilt and body sway data were chosen randomly (with replacement) and used to compute an FRF from which model parameters could be derived. Within-subject 95% confidence intervals (CIs) were calculated by repeating this procedure 400 times and selecting the 10th and 390th parameter values (sorted by size) as the upper and lower bounds. We then averaged the within-subject 95% CIs across all participants for each parameter, condition, and model variant.

#### Balance control model quality

The ability of each model variant to account for experimental FRFs (i.e., the quality of each model fit) was assessed with the Bayesian Information Criterion (BIC). Given that the models varied in complexity (M1-M4 containing 4, 5, 5, and 6 fit parameters, respectively), the BIC was used to determine whether the model fit quality was improved with additional parameters and low-frequency data points. BIC was given by:

4 $$BIC=\text{k}\cdot \text{ln}\left(n\right)-2\cdot \text{ln}(L)$$

where *k* is the number of estimated parameters in the model, *n* is the number of frequency points in the FRF, and *L* is the log-likelihood derived from the objective function *err* obtained in Eq. (3) and assuming a Gaussian distribution of errors. BIC values were calculated for each participant and stimulus condition using the parameters identified in the optimization procedure. Lower BIC values indicate a preferred model, balancing goodness of fit and model simplicity.

#### Model and parameter recovery analysis

A model recovery analysis was performed on simulated data to ensure the model variants are distinguishable^26^. First, 1000 simulated FRFs were generated for each variant in both short-PRTS and long-PRTS conditions. Anthropometric parameters used in all simulations were determined based on the mean height and weight of the participant sample. The remaining parameter values used to generate each simulated FRF varied randomly within the range of parameter values identified from our experimental data. Each simulated FRF was fit with all the model variants, and BIC scores were calculated (Eq. 4) to compare model performance. The percentage of instances in which each model variant provided the best fit to the simulated FRFs from each stimulus condition is summarized in confusion matrices. Additionally, a parameter recovery analysis was performed to confirm that the parameters of each variant are distinguishable. Similar to the model recovery analysis, 1000 simulated FRFs were generated per stimulus condition with all model variants using randomized parameter values. Each set of simulated data was fit with its origin variant, and parameter estimation was performed to ‘recover’ the model parameters used to generate each simulated FRF. Pearson’s correlation was employed to quantify the relationship between the recovered model parameters estimated from each simulated FRF and the actual model parameters used to generate the simulated FRFs.

### Statistical analysis

Statistical analyses were performed using JASP software version 0.18.1.0^27^. Means and standard deviations were calculated for model parameters and model quality measures. Model parameters and their bootstrapped CIs were compared across stimulus conditions using paired-sample t-tests. Using repeated measures analysis of variance (ANOVA), BIC values were compared across model variants and stimulus conditions. Significant interactions were followed up by examining the effect of model variant with planned Helmert contrasts separately for each stimulus condition. *P* < 0.05 was considered statistically significant.

## Results

### Experimental sway characteristics

**Fig. 2:**
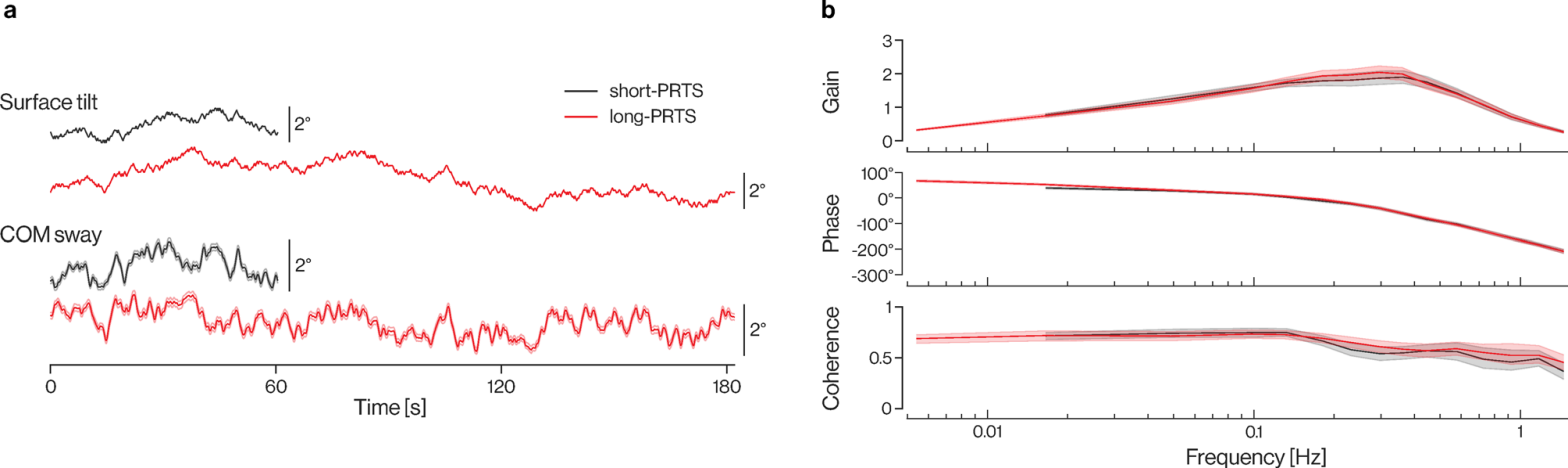
Average stimulus-evoked sway. (**a**) Time series stimulus and COM response traces in short-PRTS (black) and long-PRTS (red) conditions. (**b**) Gain (top row), phase (middle row), and coherence (bottom row) of stimulus-evoked sway. Group averages are represented by solid lines, and 95% CIs are represented by shaded areas.

Figure 2a shows the average COM sway time series, and Fig. 2b shows the experimental FRFs in each stimulus condition. Sway patterns were consistent with previous reports of experimental body sway responses to support surface tilts^1^. Gain values were highest in the 0.2–0.5 Hz range and decreased with increasing stimulus frequencies, while the phase was approximately 0° around 0.1 Hz and decreased with increasing frequencies. Coherence was also highest at stimulus frequencies between 0.05 and 0.1 Hz and decreased with increasing frequencies. At lower frequencies (~ 0.0165–0.1 Hz), a gain decline and a phase advance were observed in the average experimental FRFs. This pattern was consistent across both stimulus conditions. At the lowest frequency point, only contained in the 182-s long cycles, gain declined further, while the phase lead slightly increased. Coherence remained relatively constant between 0.0055 and 0.05 Hz, indicating that the ratio of stimulus-evoked sway to random sway did not change across these frequencies.

### Simulated FRFs

**Fig. 3:**
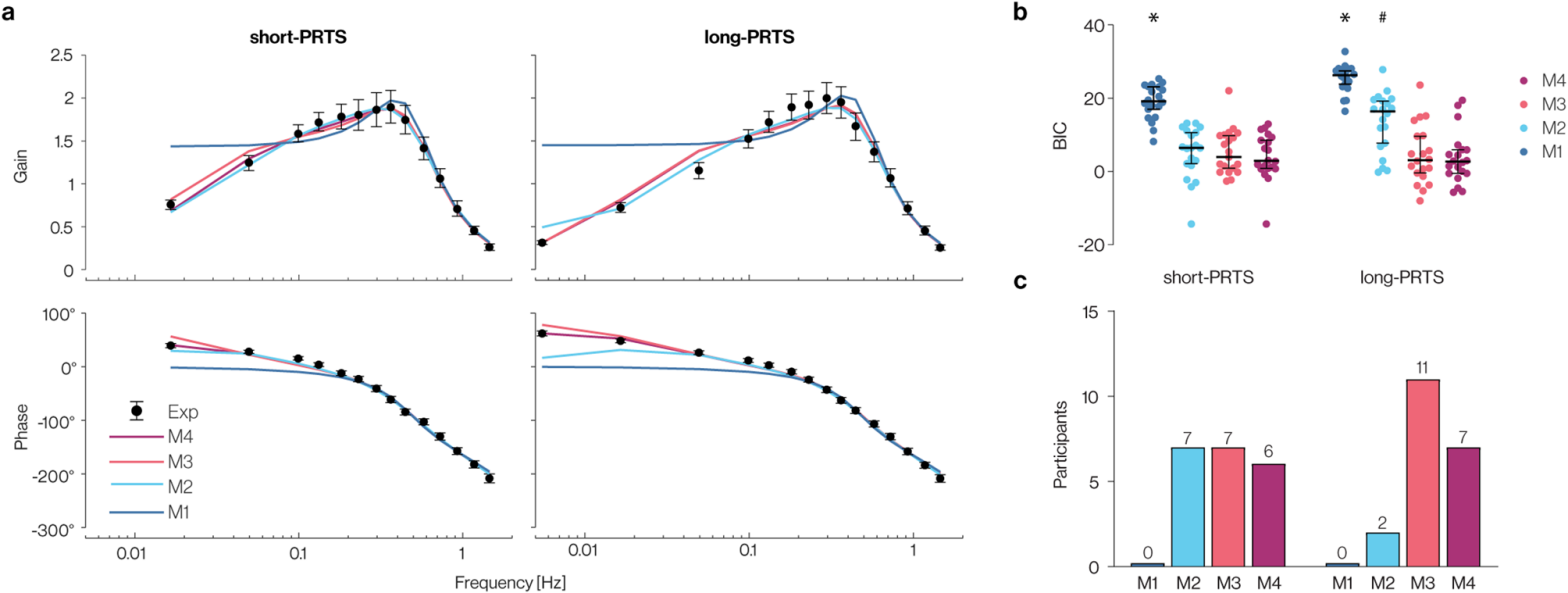
(**a**) Simulated and experimental frequency response functions. Experimental sway response means ± 95% CIs are represented by black circles with error bars, and solid color lines represent simulated sway responses. (**b**) Model quality results. Individual participant (n = 20) BIC values are shown for all model variants and stimulus conditions. Solid lines in the boxplots indicate the median, and whiskers indicate interquartile ranges. *indicates a significant (*p* < 0.05) difference from M2-M4, and # indicates a significant difference from M3-M4 based on planned Helmert contrasts. (**c**) Participants best fit by each model. Histogram demonstrating the number of participant responses best fit by each model variant across stimulus conditions. The best fit was determined by identifying the model variant with the lowest BIC when fit to each participant’s experimental FRFs.

Figure 3a shows the FRFs of the best fit of each model variant together with the experimental data. Above ~ 0.2 Hz, simulated FRFs from all model variants generally fit well with experimental data. Below 0.1 Hz, M1 diverged from experimental data, showing a constant gain and phase. M2, M3, and M4, in contrast, showed a gain decline and phase lead below 0.3 Hz in both stimulus conditions, which was in close correspondence with the experimental FRFs. In the long-PRTS condition, there were two smaller deviations from experimental results; between ~ 0.2 and 0.3 Hz, simulated values were slightly smaller, and at ~ 0.5 Hz, slightly higher compared to experimental results in all models.

The long-PRTS trials (182 s duration) added one frequency point at 0.0055 Hz compared to the short-PRTS (60.5 s duration) condition. The model performance differed considerably at this frequency point depending on the variant of the low-frequency component. The experimental phase lead increased slightly at the lowest frequency point compared to the phase at 0.0165 Hz. This behavior was reproduced by M3 and M4, while M2 showed a decline in phase. M4 showed the best match with the experimental behavior, while M3 showed a larger increase in the phase lead compared to the experimental results. In M2, both phase and gain diverged from the experimental FRF at 0.0055 Hz, with the phase lead being much smaller and the gain higher.

### Quantitative model comparison

Figure 3b shows the BIC results across model variants and stimulus conditions. A significant interaction effect between the model variant and stimulus condition was observed for BIC scores (*F*_(2.04, 38.80)_ = 18.82, *p* < 0.001, *η*^*2*^_*p*_ = 0.498). BIC scores for M1 were significantly higher than M2-M4 in the short-PRTS condition (*p* < 0.001) and long-PRTS condition (*p* < 0.001), indicating that M1 was the lowest-quality model. In the short-PRTS condition, there was no significant difference between M2, M3, and M4. However, in the long-PRTS condition, BIC scores were significantly greater for M2 than for M3 and M4 (*p* < 0.001), indicating a lower-quality model. There was no significant difference in BIC scores between M3 and M4 in the long-PRTS condition.

Figure 3c shows the number of participants with the lowest BIC scores across model variants. While M2 provided the best fit for 35% of participant responses in the short-PRTS condition, it was the best fit for only 10% in the long-PRTS condition. In contrast, M3 and M4 together best fit 65% of participant responses in the short-PRTS condition and 90% in the long-PRTS condition, with M3 accounting for most (55%) participant responses in the long-PRTS condition. None of the participants were best fit by M1 in either stimulus condition.

### Model recovery

**Fig. 4:**
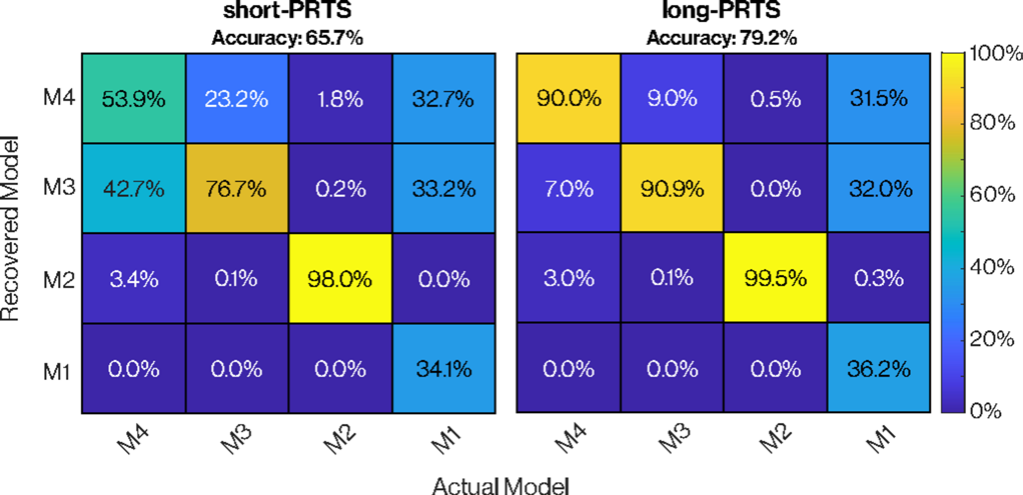
Model recovery confusion matrix. Actual model variant used to generate simulated FRFs (x-axis) compared to those that best fit simulated data (y-axis) for both short-PRTS (left) and long-PRTS (right) conditions. The value in each box represents the percentage of instances where the recovered model had the lowest BIC based on data from the actual model. The diagonal values represent the percentage of successful model recovery, while the off-diagonal values point to potential model confounding, indicating cases where one model may fit better than another. The overall accuracy was determined by calculating the average of all diagonal elements in the confusion matrix.

In this pure simulation analysis step, we tested whether simulations from a model variant were also recovered from the fitting procedure as the model with the lowest BIC score. Discrimination of model variants in the short-PRTS condition showed excellent recovery for M2 and M3 but poor recovery in M1 and M4 (Fig. 4). Specifically, simulations generated by M1 were better fit by either M3 or M4 in just under 70% of all simulations for both stimulus conditions. Furthermore, simulations generated by M4 were only slightly better fit by M4 (53.9%) than M3 (42.7%) in the short-PRTS condition. Recovery was better for all variants, particularly M4, in the long-PRTS condition compared to the short-PRTS condition.

### Model-independent parameters

Model-independent parameter estimates for M1-M4 are presented in Table 1. Parameter estimates were highly correlated across model variants (Pearson *r*’s = 0.88–1.00), and the effects of stimulus condition are similar for all model variants unless stated otherwise. *Kd* was smaller in the long-PRTS condition than in the short-PRTS condition, which was significant for all model variants except for M2. *Kp* was significantly larger in the long-PRTS condition than the short-PRTS condition for M4 but did not change for the other model variants. *Td* and *Wprop* did not significantly change across stimulus conditions. The 95% CIs for all model-independent parameters were smaller in the long-PRTS condition compared to the short-PRTS condition (Table 2). The effects reported in Table 2 were consistent across all model variants except for the 95% CIs for *Kp*, which were not significantly different between stimulus conditions for M1 and M3.

**Table 1:** Means and effect sizes of model-independent parameters.

Parameter	Condition	M1		M2		M3		M4	
		Mean (SD)	d	Mean (SD)	d	Mean (SD)	d	Mean (SD)	d
Kp [/mgh]	short-PRTS	1.53 (0.15)	− 0.05	1.48 (0.12)	− 0.21	1.46 (0.12)	0.14	**1.42 (0.09)**	0.49*
	long-PRTS	1.53 (0.16)		1.47 (0.13)		1.47 (0.14)		**1.45 (0.13)**	
Kd [/mgh]	short-PRTS	**0.43 (0.06)**	− 0.48*	0.46 (0.06)	− 0.44	**0.45 (0.05)**	− 0.60*	**0.46 (0.05)**	− 0.85**
	long-PRTS	**0.42 (0.05)**		0.45 (0.06)		**0.44 (0.05)**		**0.44 (0.05)**	
Td [s]	short-PRTS	0.15 (0.02)	0.02	0.16 (0.02)	− 0.17	0.15 (0.02)	− 0.01	0.16 (0.02)	− 0.14
	long-PRTS	0.15 (0.01)		0.16 (0.01)		0.15 (0.01)		0.15 (0.01)	
Wprop	short-PRTS	0.50 (0.06)	− 0.06	0.46 (0.06)	− 0.25	0.46 (0.06)	− 0.02	0.46 (0.06)	− 0.10
	long-PRTS	0.49 (0.06)		0.45 (0.05)		0.46 (0.06)		0.46 (0.06)	

Mean and standard deviation values for each model-independent parameter across model variants and stimulus conditions.

Bold values indicate significant comparisons between short-PRTS and long-PRTS conditions, with effect size (Cohen’s *d*) shown. *p < 0.05, **p < 0.01.

**Table 2:** Parameter confidence intervals (95%) from short-PRTS and long-PRTS trials.

Parameter	short-PRTS	long-PRTS	Effect size
Model-independent parameters*			
*Kp* [/mgh] CI	**0.128 (0.068)**	**0.099 (0.057)**	− **0.667 [**− **1.146 **− **0.173]**
*Kd* [/mgh] CI	**0.049 (0.019)**	**0.035 (0.020)**	− **0.577 [**− **1.045 **− **0.096]**
*Td* CI	**0.019 (0.009)**	**0.014 (0.005)**	− **0.639 [**− **1.115 **− **0.150]**
*Wprop* CI	**0.064 (0.028)**	**0.047 (0.015)**	− **0.720 [**− **1.206 **− **0.219]**
**Model-dependent parameters**			
*Ki*_*2*_ [/mgh] CI	**0.089 (0.071)**	**0.068 (0.052)**	− **0.546 [**− **1.010 **− **0.069]**
*Kt*_*3*_ [x1000] CI	**2.427 (1.363)**	**1.706 (0.890)**	− **0.565 [**− **1.031 **− **0.085]**
*Kt*_*4*_ [x1000] CI	**3.525 (2.129)**	**2.332 (2.088)**	− **1.028 [**− **1.564 **− **0.474]**
*Tt*_*4*_ CI	131.227 (110.544)	146.263 (89.978)	0.210 [− 0.236 0.651]

95% confidence intervals (CIs) are presented as mean (standard deviation) across single-subject CI estimates. Bold values indicate significant comparisons between short-PRTS and long-PRTS conditions at *p* < 0.05, with effect size (Cohen’s *d*) and 95% CIs for Cohen’s *d* shown in the last column. *Only estimates from M4 are shown for model-independent parameters.

### Model-dependent parameters

**Fig. 5:**
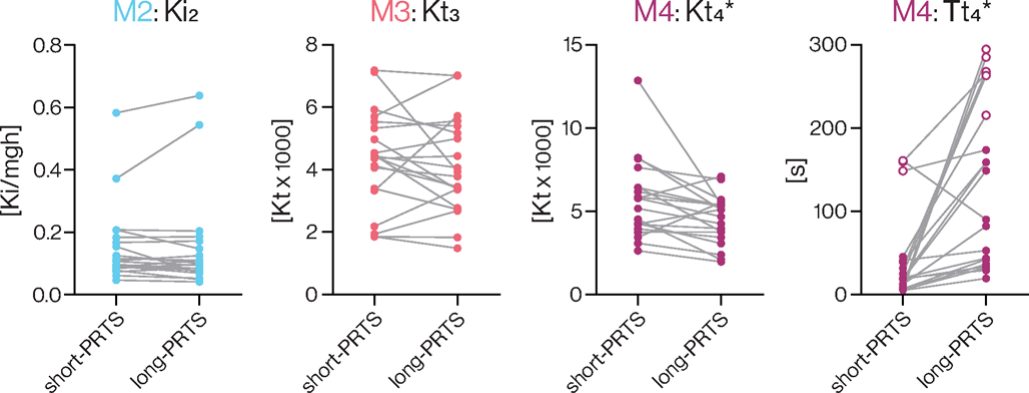
Model-dependent parameter estimates. Individual participant (n = 20) data points in each stimulus condition are connected by solid grey lines. Estimates of the M4 torque feedback time constant (*Tt*_*4*_) greater than the stimulus sequence duration in each condition (> 60.5 s in the short-PRTS condition and > 182 s in the long-PRTS condition) are represented by open circles. Significant differences between stimulus conditions (paired samples t-tests) are represented by a star (*).

Figure 5 shows the single-participant changes of model-dependent parameters of the low-frequency component in each model. The integral gain of M2 (*Ki*_*2*_, *p* = 0.947; Cohen’s *d* = 0.015) and torque feedback gain of M3 (*Kt*_*3*_, *p* = 0.407; Cohen’s *d* = -0.190) did not significantly change between stimulus conditions. Conversely, we found large differences in M4 with a smaller gain (*Kt*_*4*_, *p* = 0.020; Cohen’s *d* = − 0.568) and a larger time constant (*Tt*_*4*_, *p* < 0.001; Cohen’s *d* = 0.928) in the long-PRTS condition compared to the short-PRTS condition (Fig. 5). We then analyzed the 95% CIs of the model-dependent parameter estimates (Table 2). Overall, 95% CIs were smaller in the long-PRTS condition compared to the short-PRTS condition, indicating more accurate parameter estimates with longer stimulus periods. However, the 95% CI for *Tt*_*4*_ was larger in the long-PRTS condition compared to the short-PRTS condition, although this did not reach statistical significance. Thus, the accuracy of M4 time constant parameter estimates did not increase with longer stimulus periods.

### Parameter recovery

**Fig. 6:**
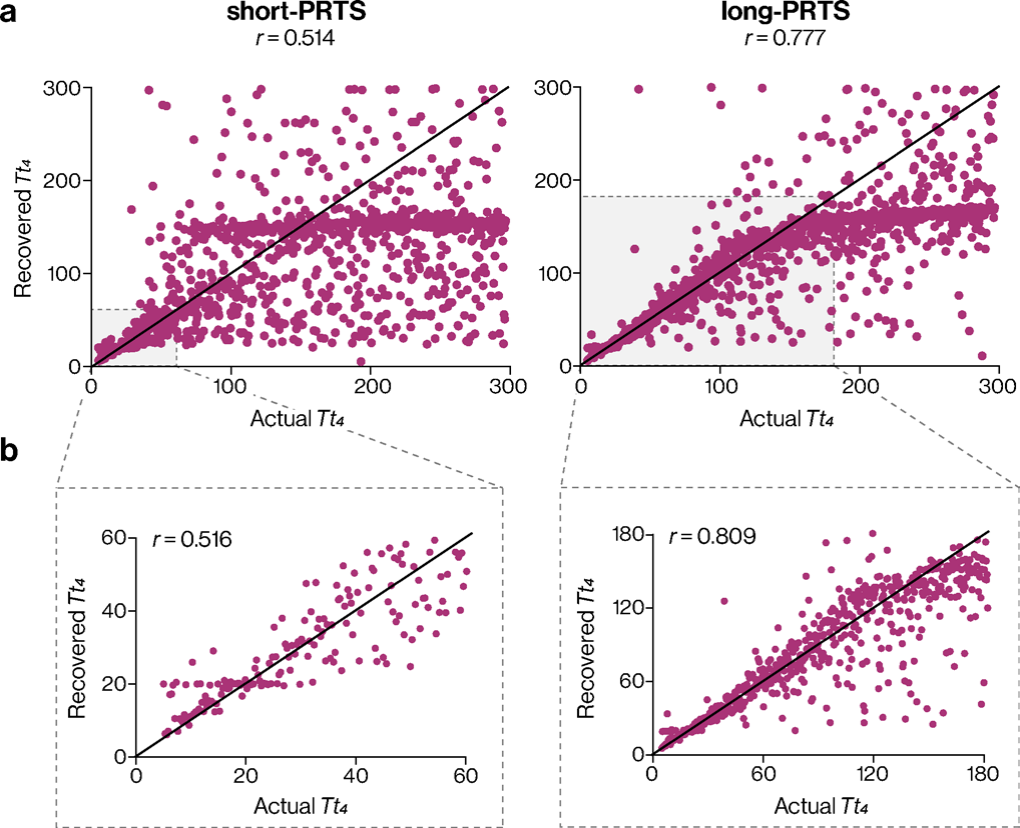
Torque feedback time constant parameter recovery. (**a**) Actual parameter values used to generate simulated FRFs (x-axis) compared to those recovered by model fitting (y-axis) for both short-PRTS (left) and long-PRTS (right) conditions. (**b**) Parameter recovery for time constant values within the stimulus sequence duration of each condition. Pearson’s correlation coefficient is shown at the top of the figure in each panel. The solid diagonal line in each panel represents perfect parameter recovery (i.e., Pearson’s correlation coefficient of 1).

Another simulation-based analysis step was performed to determine the extent to which known parameters were recovered from a fit of the simulated sway trajectories. All parameters across model variants and stimulus conditions demonstrated excellent recovery (Pearson’s *r* values range: 0.952–0.999; Supplementary Table S1) except for the M4 variant torque feedback time constant, *Tt*_*4*_. The torque feedback time constant recovery was relatively poor in the short-PRTS condition (*r* = 0.514) but improved in the long-PRTS condition (*r* = 0.777). In both conditions, torque feedback time constants below ~ 20 s were recovered relatively well, but longer time constants had poor recovery (Fig. 6a). Recovery was similar when examining torque feedback time constant values within the stimulus sequence duration in the short-PRTS (< 60.5 s; *r* = 0.516) and long-PRTS (< 182 s; *r* = 0.809) conditions (Fig. 6b). Interestingly, simulated FRFs with torque feedback time constants greater than ~ 80 s and ~ 110 s were frequently recovered as ~ 150 s.

## Discussion

The current study investigated the slow dynamics of human balance control using long-duration support surface tilt sequences. Our results provide strong evidence that human balance control contains a feedback mechanism attenuating sway at frequencies < 0.1 Hz. Model M1 does not contain such a slow mechanism and strongly deviated from experimental sway responses < 0.1 Hz, consistent with earlier findings^6^. We further compared three variants: a controller with an integral term (i.e., a PID controller) and two variants of a positive torque feedback loop. Our results demonstrate that the two model variants with torque feedback best fit experimental data, especially for the long-duration stimulus sequences. This supports the hypothesis that positive torque feedback contributes to anteroposterior balance control. However, questions remain regarding the time horizon of this torque feedback, which was very difficult to estimate.

The torque feedback mechanism is thought to provide a reference signal for upright standing^3^. Using force/torque cues as a reference for standing provides a spatial anchoring against which noisy kinematic sensory cues can be self-calibrated, mitigating drift and system instability. This also allows the balance control system to align the body with an orientation associated with minimal torque output, given the specific environmental and task constraints^7^. In this way, the torque feedback mechanism reduces the effort required to stand. This implies that the reference for upright standing is not a fixed position in space, but rather a slowly updating, dynamic reference that accounts for the body configuration and its relation to gravity. Previous research has hypothesized the existence of a slowly fluctuating reference around which body oscillations are controlled^28–30^. This was highlighted by the observation that individuals, following an ultraslow toes-down tilt of the support surface, gradually return toward an upright stance but maintain a slightly forward-leaning position^29^. If participants used the gravitational vertical as a reference, they would return to the original upright position. Likewise, several studies have reported that individuals gradually return to a normal slight forward lean following extended periods of standing on an inclined surface^31–33^. We propose that the abovementioned observations can be attributed to the torque feedback mechanism.

Torque feedback requires that the central nervous system encodes a signal that is equivalent to, or associated with, the COP^34,35^. Information related to the COP likely arises from a combination of cues signaling the length and/or force generated by the foot and lower leg muscles, the relative position of body segments, and support surface interactions. The source of such signals could arise from plantar mechanoreceptors sensing pressure under the feet^36–38^, Golgi tendon organs in the ankle plantar flexors^39,40^, or intrinsic muscle spindles in the foot^41^. Another important consideration is the neurophysiological mechanism by which an encoded COP signal might be low-pass filtered. Although the exact process remains unclear, evidence shows that Golgi tendon organs encode force across a broad frequency range^42^, implying that a low-pass filtering likely arises from central processing mechanisms.

Estimates of balance control model parameters are expected to become more precise with increased stimulus durations and a greater number of stimulus sequences^17,43,44^. Our findings corroborate this for model parameters related to mid-high frequency behavior (Supplementary Table S2) and for most parameters associated with low-frequency behavior. Despite the long trials, the time constant of the low-frequency component of human balance control showed large intra- and inter-individual variability, reflected in very large confidence bounds in both stimulus conditions. It is unclear why some estimates of the torque feedback time constant exceeded the duration of both stimulus sequences. To explore this further, we repeated the parameter estimation procedure while constraining the time constant estimates by matching the upper bound of the global optimization to the duration of the stimulus sequence in each condition. This approach resulted in many boundary solutions whereby the best estimate for the time constant reached the upper bound of the defined parameter range. The poor recovery of the time constant parameter beyond ~ 2/3 of each stimulus sequence duration (~ 40 s in the short-PRTS condition and ~ 120 s in the long-PRTS condition; see Fig. 6) suggests that the experimentally identified large time constant values may be an artifact of instability in the estimation procedure. In a more general sense, this may also indicate that long experimental trials offer limited advantages for certain parameters associated with the slow dynamics of balance control. However, the large confidence bounds we found for *Tt*_*4*_ were likely inflated by the larger values of the time constant itself. When expressed as a percentage of the time constant value (i.e., (CI / *Tt*_*4*_) * 100), the torque feedback time constant 95% CIs improved considerably from 489 ± 471% in the short-PRTS condition to 139 ± 97% in the long-PRTS condition. Thus, in relative terms, the estimate did become more accurate with longer trials.

Nonetheless, while M4 provided the most accurate representation of behavior in long-duration trials, our results indicate the torque feedback models are poorly distinguished from each other in the short-PRTS condition, suggesting a marginal benefit of including the time constant parameter when modelling balance responses to stimulus sequences shorter than 60 s. Excluding the time constant parameter reduces the number of parameters to be estimated, thus mitigating the risk of overparameterization or parameter interactions that inversely affect simulated FRFs^5^.

Several additional aspects related to the slow dynamics of sway behavior should be considered. First, the behavior at these very low frequencies might not be time-invariant. Previous experiments have noted the high variability observed when estimating the torque feedback time constant^12^. It is possible that changes in behavior, such as set-point shifts (i.e., a participant deciding to lean a little more in one direction), may have occurred during longer trials, leading to high inter-trial variability. Observations of high variability in low-frequency sway during quiet standing^45^ could indicate such set-point shifts but likely include considerable low-frequency noise content. Second, the balance control strategy employed by participants may have varied between days. Previous studies have shown that estimates of the torque feedback gain had poor to moderate reliability across days when derived from 15 visual scene tilt sequences in virtual reality^11^. Third, the relatively poor recovery of the torque feedback time constant (Fig. 6) raises the possibility that the M4 variant is not properly specified, and some other experimental approach and/or model structure may be required to explain the distinct balance behavior observed at low frequencies. Finally, our experiment focused exclusively on anteroposterior sway and balance control through ankle plantarflexion/dorsiflexion torques; thus, it remains unclear whether this mechanism extends to mediolateral balance control.

In summary, we provide further evidence that humans use torque cues from the feet and legs in a positive feedback mechanism during standing to remain upright. Some of the properties of this mechanism were difficult to estimate even with long-duration trials, highlighting the trade-off between parameter accuracy and stimulus duration in systems identification. Detailed information about the torque feedback mechanism might be revealed using a different experimental approach^46^. The variability observed in the properties of the torque feedback mechanism may provide valuable insights into individual differences in self-calibration and could have significant implications for understanding balance deficits.

## Electronic supplementary material

Below is the link to the electronic supplementary material.

Supplementary Material 1

## Data Availability

The experimental data that support the findings of this study are available in Figshare with the identifier https://doi.org/10.6084/m9.figshare.28346933.
