# Supplementary material for: Slow dynamics of human balance control
Source: Sci Rep. 2025 Jul 29;15:27593. doi: 10.1038/s41598-025-09392-2 (PMC12307746; doi:10.1038/s41598-025-09392-2)
Supplement: Supplementary file 1 — Supplementary Material 1 [file 41598_2025_9392_MOESM1_ESM.pdf]

Supplementary Material

Table S1. Parameter recovery for all model variants.

Parameter	M1		M2		M3		M4	
	short-PRTS	long-PRTS	short-PRTS	long-PRTS	short-PRTS	long-PRTS	short-PRTS	long-PRTS
Proportional gain [*Kp*]	0.999	0.999	0.997	0.998	0.993	0.996	0.995	0.994
Derivative gain [*Kd*]	0.997	0.997	0.981	0.995	0.992	0.996	0.993	0.990
Time delay [*Td*]	0.993	0.994	0.954	0.987	0.987	0.993	0.989	0.984
Proprioceptive weight [*Wprop*]	0.997	0.997	0.952	0.995	0.993	0.996	0.981	0.986
Integral gain [*Ki*]	-	-	0.996	0.996	-	-	-	-
Torque feedback gain [*Kt*]	-	-	-	-	0.992	0.991	0.993	0.992
Torque feedback time constant [*Tt*]	-	-	-	-	-	-	0.514	0.777

Note: Pearson correlation coefficients are shown for the relationship between simulated and recovered parameter values across all model variants and stimulus conditions. Parameter values used in the simulations were randomly selected from the range of parameter values obtained from fitting experimental data.

Table S2. Model-independent parameter confidence intervals (95%) for all model variants.

		M1		M2		M3		M4	
**Parameter**	**Condition**	**Mean (SD)**	**d**	**Mean (SD)**	**d**	**Mean (SD)**	**d**	**Mean (SD)**	**d**
Kp	short-PRTS	0.110 (0.059)	-0.414	0.107 (0.056)	**-0.601**	0.106 (0.045)	-0.395	0.128 (0.068)	**-0.667**
	long-PRTS	0.087 (0.040)		0.081 (0.040)		0.090 (0.039)		0.099 (0.057)	
Kd	short-PRTS	0.050 (0.020)	**-0.647**	0.050 (0.019)	**-0.510**	0.049 (0.020)	**-0.588**	0.049 (0.019)	**-0.577**
	long-PRTS	0.035 (0.022)		0.036 (0.021)		0.036 (0.020)		0.035 (0.020)	
Td	short-PRTS	0.018 (0.008)	**-0.686**	0.018 (0.009)	**-0.616**	0.018 (0.008)	**-0.563**	0.019 (0.009)	**-0.639**
	long-PRTS	0.013 (0.005)		0.014 (0.005)		0.014 (0.005)		0.014 (0.005)	
Wprop	short-PRTS	0.076 (0.031)	**-0.985**	0.065 (0.028)	**-0.616**	0.064 (0.026)	**-0.727**	0.064 (0.028)	**-0.720**
	long-PRTS	0.050 (0.016)		0.049 (0.017)		0.048 (0.015)		0.047 (0.015)	

Note: Mean (standard deviation) presented for each estimated parameter 95% confidence interval. The effect (Cohen's d) of stimulus conditions is also shown. Bold values indicate significant comparisons between short-PRTS and long-PRTS conditions at $p < 0.05$. See text for model variant details.
